# Case report: apixaban‐induced thrombocytopenia

**DOI:** 10.1002/ccr3.809

**Published:** 2017-02-01

**Authors:** Oliver Snellgrove

**Affiliations:** ^1^ Intensive Care UnitHornsby HospitalHornsbyNew South WalesAustralia

**Keywords:** Apixaban, thrombocytopenia

## Abstract

Apixaban is a relatively new drug, and its safety profile is still being established. Thrombocytopenia is an uncommon, but life‐threatening side effect with no previous published case repots. Clinicians must be aware of this and exercise caution particularly as some studies hint that it may be used in the management of HIT.

## Introduction

Prevention of nonvalvular atrial fibrillation is increasingly reliant on new anticoagulants that do not require regular monitoring. Apixaban has shown superiority in the prevention of stroke or systemic embolism versus warfarin with decreased risk of bleeding 2 and as such is being increasingly prescribed. Apixaban has a fast onset of action acting as a direct inhibition of factor Xa.

We report a case of possible apixaban‐induced thrombocytopenia, which although has been reported as a potential side effect in the product monograph 1, no case reports have been identified in the literature.

## Case Report

A 78‐year‐old male was admitted to the intensive care unit with severe thrombocytopenia (platelet count of 1 × 10^9^/L) and congestive heart failure while on apixaban for atrial fibrillation. Apixaban was commenced 4 months prior to his presentation. Routine blood tests prior, as well as 2 months after, demonstrated a normal platelet count of 312 and 301 × 10^9^/L, respectively. Prior to his admission, he had been taking diuretics including furosemide which had recently been decreased.

In the Emergency Department, he was tachypnoeic with type 1 respiratory failure and was placed on noninvasive ventilation. He received ceftriaxone, azithromycin, and one pack of pooled platelets, as well as an additional dose of furosemide. He was admitted to the intensive care unit, and the following morning his platelet count had risen to 36 × 10^9^/L. At the time of admission, he had a disseminated intravascular coagulopathy (DIC) screen sent as well as an autoimmune screen, and abdominal ultrasound, which showed no evidence of splenomegaly, TTP, or DIC. Hematology was consulted who agreed the most likely cause of the thrombocytopenia was drug‐induced. He had been started on empirical antibiotics for a possible underlying pneumonia in the Emergency Department which was ceased the following morning. An atypical pneumonia screen was sent which was negative for pneumococcus, chlamydia, mycoplasma, and legionella. All blood cultures were negative. In addition, viral PCR swabs were taken which all returned negative. His initial inflammatory markers demonstrated a WCC of 12 × 10^9^/L which remained stable for his entire admission. During his admission, no convincing evidence for infection was obtained either clinically or biochemically.

He remained on the intensive care unit for 2 weeks managing his congestive heart failure with diuretics, inotropes, and noninvasive ventilation which gradually improved. All of his heart failure medications were increased including furosemide, and his apixaban immediately ceased.

He received one dose of ceftriaxone and one dose of azithromycin in the Emergency Department, and the only change in medication was the cessation of apixaban. Day 3 showed an improving platelet count of 42 × 10^9^/L which continued to slowly increase to normal levels over the next week. He was transferred to the ward where he remained for a further week before being discharged home without further anticoagulation.

## Discussion

Thrombocytopenia caused by apixaban has been reported in the product monograph; however, severity and incidence are not specified. To the best of our knowledge, the cause of the thrombocytopenia in the above case was a result of apixaban; however, alternative mechanisms should also be discussed.

The patient presented in respiratory distress and thrombocytopenic, so sepsis and DIC were considered to be the initial working diagnoses in the Emergency Department. As a result, antibiotics were administered as well as pooled platelets which make establishing a clear temporal relationship between ceasing apixaban and the return of a normal platelet count difficult. The DIC screen that was sent on the initial blood tests, taken before the administration of antibiotics, including fibrinogen and fibrin degradation products, was unremarkable. Despite this, we are unable to completely exclude infection as a cause as the patient received both ceftriaxone and azithromycin both of which have relatively long half‐lives.

In addition to sepsis, furosemide itself has been described in the literature as a cause of thrombocytopenia 3. The mechanism of action for this is poorly understood and, however, appears to be dose‐dependent in the above case. Our patient had been on furosemide for over 2 years, and his dose had recently been decreased rather than increased. Although there are documented cases where furosemide can cause thrombocytopenia, it seems less likely in our patient given the recent reduction in dose and ongoing administration in hospital.

Apixaban was started 4 months prior to his presentation, and during that time period, no new medications were started. Although his platelet count was normal 2 months after starting the drug, we do not have an understanding of the exact mechanism of action and immune‐mediated drug reactions may be delayed in their onset. With no evidence to support sepsis or DIC as the cause, nor any alternative drug reactions, we are forced to consider apixaban as a cause for the thrombocytopenia.

Apixaban is an emerging drug used more commonly for the prevention of stroke and embolic events, and as its use increases, the frequency of adverse drug reactions will increase. It is important for clinicians to be aware of potential drug reactions and side effects when choosing to prescribe it but also when considering differential diagnosis when managing the patient with an unexplained illness. As mentioned above, thrombocytopenia was a recognized side effect in clinical trials. It is possible that the thrombocytopenia was unrelated to the apixaban as all side effects during clinical trials are reported; however, as it is a relatively new drug, uncommon side effects may be underreported and more case reports are needed to accurately determine the true incidence of apixaban‐induced thrombocytopenia.

## Authorship

OS: sole author

## Conflict of Interest

None declared.
